# Local and systemic factors both required for full renewal of deer antlers, and systemic factors only for generic cutaneous regenerative healing

**DOI:** 10.1186/s13619-025-00233-1

**Published:** 2025-06-10

**Authors:** Wenying Wang, Qianqian Guo, Chunyi Li

**Affiliations:** 1 Institute of Antler Science and Product Technology, Changchun Sci-Tech University, Changchun, 130000 China; 2 Jilin Provincial Key Laboratory of Deer Antler Biology, Changchun, 130000 China

**Keywords:** Wound healing, Scar, Deer antler, Pedicle, Systemic factor, Local factor, Regeneration, Bone, Pedicle periosteum, Myofibroblast transformation

## Abstract

Deer antlers are the only mammalian organs that periodically regenerate from permanent bony protuberances (pedicles). Antler regeneration relies on the presence of pedicle periosteum (PP) and starts from regenerative healing of wounds created following the hard antler casting. Interestingly, PP deletion (removal of local factors) abolishes antler regeneration and the transition to velvet skin (shiny and hair sparsely populated) but cannot inhibit regenerative wound healing although the healed tissue is of pedicle type (scalp-like); this indicates that systemic factors from circulating blood contribute to the generic regenerative wound healing. Subsequently, we created full-thickness excisional (FTE) skin wounds on the forehead region in sika deer. Different healing outcomes ensued, namely regeneration or formation of a scar, depending on whether the intervention took place during the period of antler regeneration (ARP; spring–summer) or in the period where antler regeneration does not occur (non-ARP; winter). Forehead wounds during ARP exhibited regenerative healing, whereas during the non-ARP, healing took place but with a scar. Therefore, systemic factors from the circulating blood during the ARP must be responsible for this outcome. Topical application of deer blood plasma (a source of systemic factors) from ARP to FTE wounds in rats promoted regenerative healing, whereas, that from non-ARP failed to do so. Further evaluation showed that regenerative healing was achieved through increased cell proliferation, impaired inflammatory response, reduced myofibroblast transformation, and orchestrated collagen remodeling accompanied by an increase in the ratio of TGF-β3 to TGF-β1. Comparative proteomics analysis of deer plasma identified some highly up-regulated factors from the plasma in ARP (ARPP) with regeneration-stimulating-potential, such as IGF1 and PRG4. Topical application of IGF1 or IGF1 + PRG4 to rat FTE wounds greatly promoted regenerative healing; particularly in the combination of IGF1 and PRG4 group. In summary, our findings convincingly demonstrate that the systemic factors from deer blood taken during ARP contain factors capable of inducing regenerative wound healing and that this effect is not species-specific. Because there are almost no restrictions on the supply of deer blood in ARP, our findings have laid the foundation for devising effective therapies for scar-less wound healing in the clinical setting.

**Supplementary Information:**

The online version contains supplementary material available at 10.1186/s13619-025-00233-1.

## Background

**Fig. 1:**
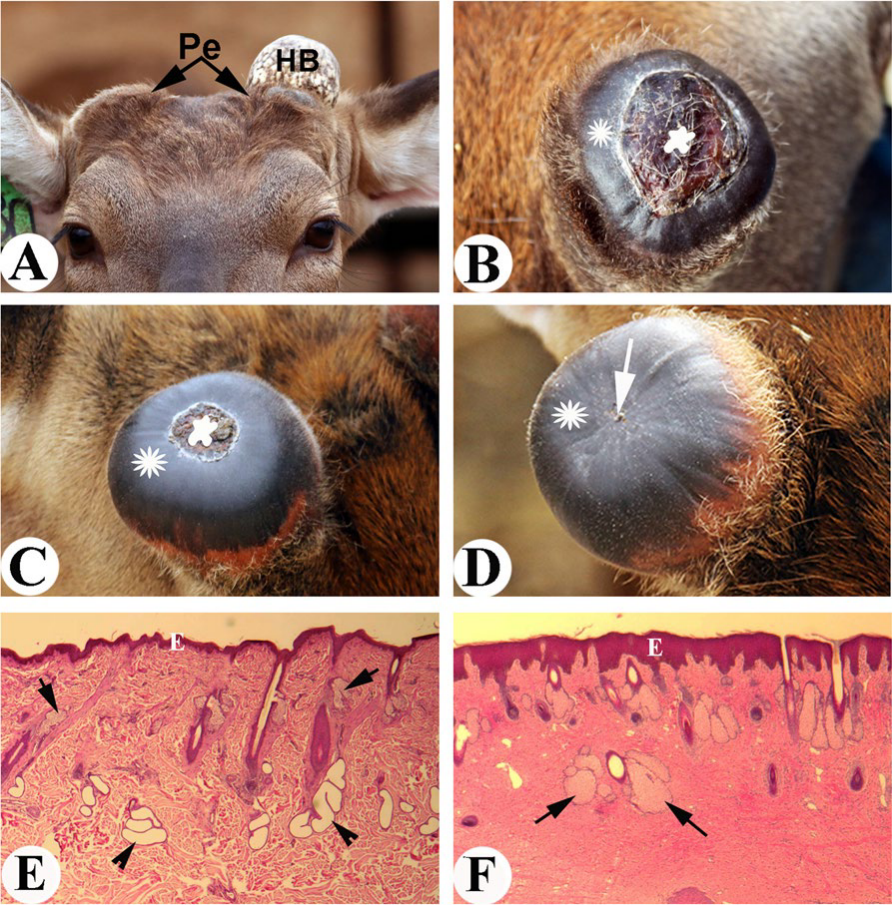
Pedicle wound healing and histological structures of pedicle skin and antler velvet skin. **A**. Pedicles (Pe) of a 3-year-old sika deer. Note that hard antler button (HB) at right side has cast and at left side still remains, which will cast within hours. **B**. Wound healing status over a pedicle stump on the 3rd day of HB casting. Note that a ring of shiny black color skin (asterisk) of a centimeter width emerged, which was circling the central scab region (star). **C**. Wound healing status over a pedicle stump on the 6 th day of HB casting. Note that shiny black color skin ring had significantly migrated centripetally (asterisk), and had substantially encroached the scab area (star). **D**. Wound healing status over a pedicle stump on the 10^th^ day of HB casting. Note that the healing shiny black color skin (asterisk) had converged in the centre (arrow), indicating the completion of wound healing. **E**. Histological structure of pedicle skin. Note that there is no visible difference from scalp skin/other somatic skin, and it contains richly distributed sweat glands (arrowhead), thin epidermis (**E**), large hair shafts with small size sebaceous glands (arrow), and arrect pilli muscle. **F**. Histological structure of velvet skin. Compared with pedicle skin, velvet skin is adorned with extremely exaggerated multi-lobed sebaceous glands (arrow), absence of sweat glands and arrect pilli muscle; has hair follicles at various developmental stages and thickened epidermis (**E**)

Deer antlers are the only mammalian appendages that not only fully regenerate but do so repeatedly on a yearly basis (Goss 1983). Each year, in late spring or early summer, totally calcified antlers or hard buttons (if growing antlers are removed at their growth phase for traditional medicine) are cast from permanent bony protuberances (Fig. 1A), known as pedicles; antler regeneration then immediately takes place from the wound healing of a pedicle stump in most deer species; while in the regenerating phase, antlers are enveloped with a specialized velvet-like pelage, called velvet or velvet skin; in early autumn, antlers are totally calcified in response to sharply rising androgen hormones in circulation in deer prior to rut, and the velvet skin is peeled off concurrently due to occlusion of blood supply to expose bare hard antlers; in the next spring, hard antlers are cast again to trigger a new round of antler regeneration (Goss 1995; Kierdorf et al. 2003; Li et al. 2004, 2005). Because annual renewal of antlers, as an appendage, is the only case in mammals, it offers the unique opportunity to investigate the mechanism underlying mammalian organ regeneration in a natural setting.

## Pedicle wound healing is regenerative but with altered skin type (antler velvet)

Antler regeneration starts from regenerative wound healing (up to 10 cm in diameter) over the pedicle stump (Li et al. 2004, 2005). Examination of the fresh casting surface immediately after hard antler casting, an exposed depressed central wound is found to be surrounded by a rim of shiny almost-hairless skin (Fig. 1B). It is this skin that gives rise to the future antler velvet, which is readily distinguishable from the pedicle skin (typical scalp skin) from which velvet skin is transformed. As the wound healing process advances, the shiny skin ring migrates centripetally, which further reduces the central scab area (Fig. 1C), until the peripheral healing skin converges in the center of the healing top of the pedicle. Subsequently, the centrally-located scab is flaked off and only until then the regenerative healing nature is fully revealed (Fig. 1D).

Velvet skin in sika deer is very different from pedicle skin (typical scalp skin), although the former is derived from the latter: shiny black color and hairs are hardly seen as they are very thin and sparsely populated (Fig. 1C, D). Histologically (Li 2010; Li and Suttie 2000), pedicle skin is no different from scalp skin and contains richly distributed sweat glands, a thin epidermis, large hair shafts and arrect pilli muscle (Fig. 1E); whereas velvet skin is adorned with very exaggerated multi-lobed sebaceous glands, is absence of sweat glands and arrect pilli muscle, has hair follicles at various developmental stages and has a thickened epidermis (Fig. 1F). Although velvet skin is unique and very different from the currently known skin types, it is bona fide skin and not a scar as it has well organized dermal and epidermal texture and contains skin appendages, albeit in a different pattern to the pedicle skin. Velvet skin is a temporary integument and naturally lasts up to 100 days; the demise of each year’s velvet skin in autumn has been considered as a case of “murder” (due to occlusion of blood vessels caused by total antler calcification), not “suicide”, since when transplanted from a growing antler either to the deer’s hind leg (Billingham et al. 1959) or to the forehead region (Goss 1972), it survived for several years.

## Local and systemic factors both are involved in regenerative wound healing, but only local factors are responsible for alteration of skin type

**Fig. 2:**
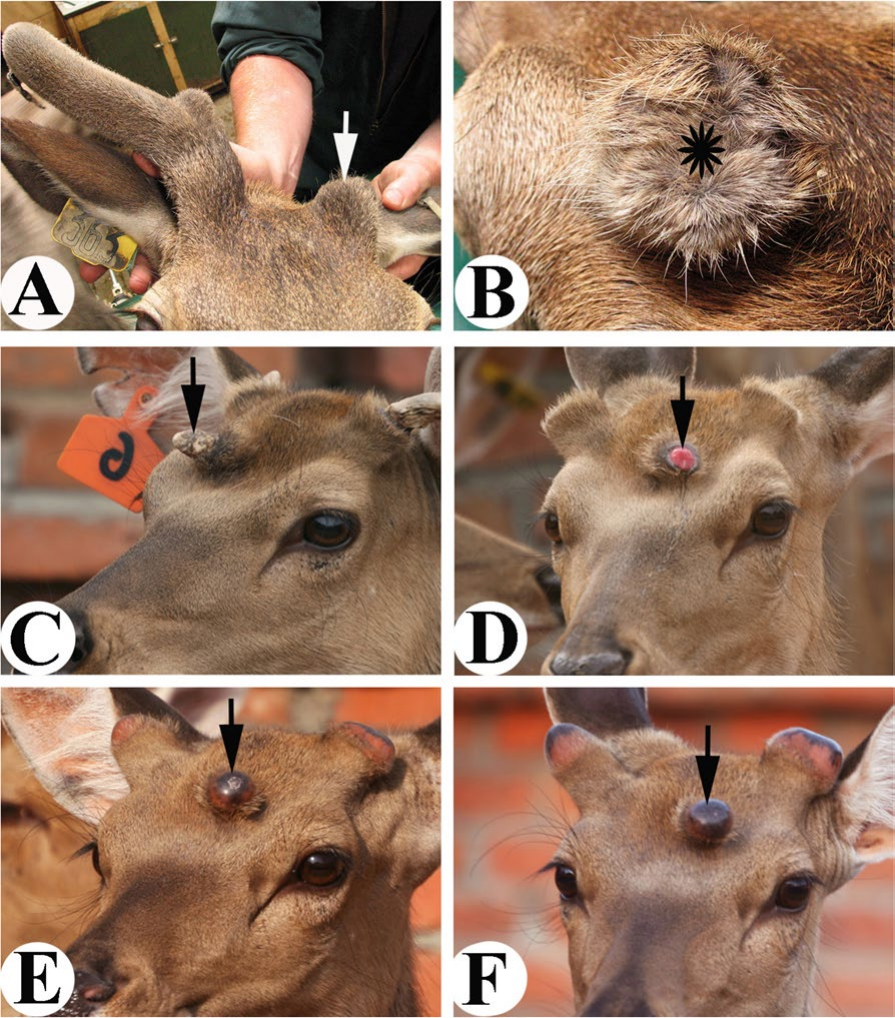
Consequences of deletion and ectopic transplantation of pedicle periosteum (PP) (**C**, **D**, **E**, **F**, reproduced with the permission from Fig. 8: a, b, c, d; Cell and Tissue Research; 10.1007/s00441-021-03505-9).** A**. Deletion of the PP from left side pedicle resulted in failure of antler regeneration from the PP-less-pedicle (arrow).** B**. Top view of the same PP-less-pedicle of Fig. A. Note that wound healing was achieved in a regenerative manner (densely populated hairs), although regenerated skin is the scalp skin type in nature (asterisk), rather than velvet type. **C**. Ectopic hard antler (arrow) on the deer forehead region generated from the transplanted antlerogenic periosteum (AP), from which PP is derived during antler ontogeny. **D**. Wound (arrow) of an ectopic pedicle stump created immediately following the ectopic hard antler casting in early spring. **E**. Healing process of the ectopic pedicle stump wound was in synchronization with the orthotopic ones, and healed skin was velvet skin type in nature (arrow).** F**. Wound healing over the ectopic pedicle stump by shiny black color skin (velvet; arrow) was completed within 10 days

Although being claimed as regenerative, the healed skin over a pedicle stump is no longer a scalp/pedicle-type in nature but a velvet-like type (Guo et al. 2021; Li et al. 2005). In an investigation of the role of pedicle periosteum (PP) in antler regeneration, we found that deletion of the PP (also means: removal of local factors) prior to antler regeneration in spring effectively abolished antler regeneration (Fig. 2A), indicating that PP is the key tissue type for annual renewal of antlers (Li et al. 2007). Based on the previous studies, PP is the tissue that is directly differentiated from the antlerogenic periosteum (AP) that forms the pedicle and first antler; and gives rise to the reserve mesenchymal (RM) cells that are responsible for rapid elongation of regenerating antlers (Feleke et al. 2021; Li and Fennessy 2021). Further characterization of the PP cells demonstrates that PP cells have stem cell attributes (Li et al. 2009b; Wang et al. 2019) and thus been called antler stem cells (AnSCs). Interestingly, PP-less pedicles successfully achieved regenerative wound healing, although the healed skin was no longer velvet-type, and instead was scalp/pedicle skin type (Fig. 2B); (Li et al. 2007). This result indicates that systemic factors in the deer’s circulating blood during the antler regeneration period (ARP) must have contributed to the regenerative healing of cutaneous wounds over the pedicles; whereas the local factors from the PP (where AnSCs reside) must be indispensable for antler regeneration, along with the effects of skin alteration including exaggerating sebaceous glands, abrogating sweat glands and arrect pilli muscle, and miniaturizing and thinning hair follicles.

To further confirm the role of local factors in velvet skin transformation during pedicle wound healing, we subcutaneously transplanted AP, a tissue that overlies the frontal crest in a male deer calf and gives rise to pedicles (including PP) and first antlers when the deer is approaching puberty (Goss and Powel 1985), to the forehead region (Guo et al. 2021). In the same year, an ectopic antler had its growth successfully initiated in the transplanted region in summer and became hard in autumn (Fig.2C) in a synchronized manner with orthotopic antlers. In the next spring, sequestration of the ectopic hard antler occurred and created a full thickness cutaneous wound on top of the ectopic forehead pedicle (Fig. 2D). This wound was subsequently healed by velvet-type skin through a regenerative manner (Fig. 2E, F). Obviously, forehead skin itself does not have the ability to transform to antler velvet. These results demonstrate that local factors from the AnSCs are capable of transforming scalp skin into velvet skin during the process of wound healing at an ectopic place (forehead), whereas absence of the local factors at an orthotopic place (apex of a PP-less pedicle) caused regenerative wound healing to be completed via pedicle/scalp skin (Fig. 2B). Therefore, the local factors from PP stimulating skin type alteration are not site-specific.

**Fig. 3:**
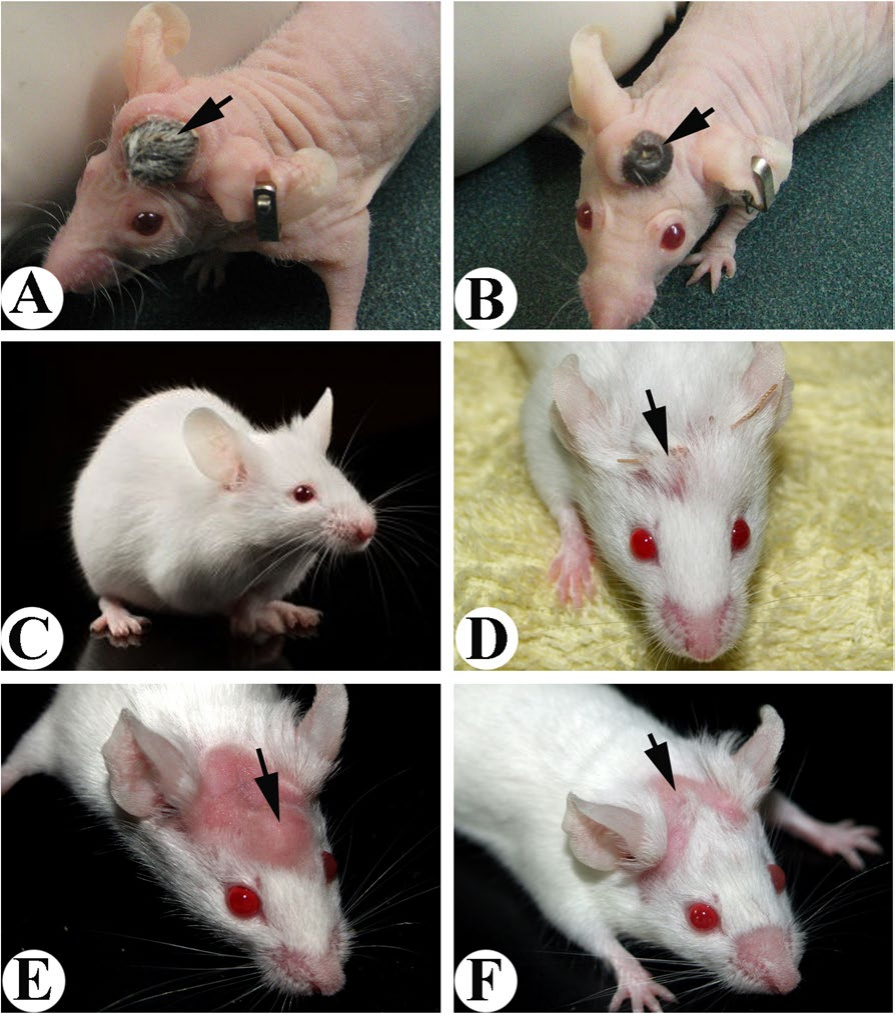
Outcome of AP xenotransplantation (Fig. A, B, D, E, reproduced with the permission from Fig. 12; BioMed Research International; http://dx.doi.org/10.1155/2013/643601).** A**. Co-transplantation of AP and deer scalp skin (arrow). Note that the transplant was survived and skin hairs started to grow.** B**. Xenogeneic antler formed from the co-transplant in Fig. 3A. Note that the co-transplanted scalp skin had transformed into velvet-like skin (arrow) completely. **C**. Kunming mouse that was used for AP transplantation. **D**. A piece of AP was transplanted subcutaneously onto the forehead of a Kunming mouse. The transplanted AP was directly under the suture (arrow).** E**. A bulge was formed from the transplanted AP. Note that the mouse skin covering the bulge was completely denuded (arrow).** F**. Hairs of the denuded area overlying the bulge were gradually growing back (arrow) when the bulge started to shrink, possibly due to the attack by the host immune system

To further support this claim, we transplanted AP together with the deer scalp skin onto the forehead of a nude mouse (Fig. 3A). Convincingly, the transplanted AP induced scalp skin to transform to velvet skin (Fig. 3B) at the nude mouse forehead region (Li et al. 2009a). Unambiguously, local factors from AnSCs are capable of transforming somatic skin into a special type of pelage, the velvet skin.

## Local factors capable of inducing regenerative wound healing do so via altered skin type, and is not species-specific

To confirm whether local factors from AnSCs inducing regenerative pedicle wound healing via altering the skin type (velvet) is species-specific, we conducted a series of experiments using a full thickness excision (FTE) wound model in rats. Firstly, we transplanted a small piece of AP subcutaneously onto the forehead regions of Kunming mice (Fig. 3C, D) and found that the overlying skin turned into apparently hair-less skin (Fig. 3E) so long as the incision suture was directly located on top of the grafted AP. Hairs of the denuded area overlying the bulge were gradually growing back (arrow) when the bulge started to shrink (Li et al. 2013). Injection of AnSCs via tail veins to rats with FTE wounds induced regenerative wound healing, and the healed skin had more richly populated large sebaceous glands, reminiscent of velvet skin, than those of intact or human-mesenchymal stem cell (hMSC)-treated skin (Rong et al. 2020) (Fig. S1). Topical application of AnSC-conditioned medium in hydrogel on the FTE wounds in rats achieved regenerative wound healing (Rong et al. 2019), and again the regenerated tissue resembled velvet skin in that more exaggerated sebaceous glands were encountered than those of hMSCs (from human umbilical cord) treated and EGF-treated skin (Fig. S1A). We (Zhang et al. 2023a) took it further via both topical application and local injection of AnSC-exosomes around the FTE wounds in rats and used AnSCs and MSCs (from rat bone marrow) as the positive controls, and found all the AnSC-exosome-treated and positive control groups achieved regenerative wound healing. However, only the healed skin from AnSC-treated and AnSC-exosome-treated groups had more exaggerated sebaceous glands than that from the rat-MSC-treated group (Fig. S1B). Therefore, the local factors from AnSCs stimulating alteration in skin type into velvet skin are not species-specific.

## Exploration of the mechanism underlying the local factor stimulation of regenerative wound healing

It is known that MSCs promote wound healing through their differentiation potential, immunomodulatory properties and paracrine effects (Seifert et al. 2012). Increasing evidence has shown that MSCs inducing wound healing is more likely to be through the paracrine pathway (Zomer and Trentin 2018). MSCs secrete bioactive molecules to modulate their microenvironment; promote angiogenesis; stimulate resident cell proliferation; promote cell migration, cell differentiation, survival and functional recovery of metabolism (Foster et al. 2021); and eventually achieve an optimal wound healing outcome (Li et al. 2009b).

The fact that paracrine factors from the MSCs play more important roles beyond direct participation of cells in cutaneous wound healing can be better illustrated in the case of AnSCs promoting scarless healing over pedicle stumps during the initial stage of antler regeneration. AnSCs reside in the PP and do not enter the overlying cutaneous compartment to participate in wound healing (Li and Suttie 2000). These results further suggest that paracrine factors from the AnSCs may be even more potent than those from other types of currently known MSCs, as the AnSC factors can traverse up to 1 mm in distance to reach and effect wound healing without requiring the AnSCs themselves to enter the healing tissue. In this respect, a distance of 1 mm can be considered as an exceedingly long distance in molecular terms. Besides offering a more efficacious therapy for wounds, the “long-distance” effects of AnSC paracrine factors on target tissues would be advantageous when a wound healing reagent is to be developed for clinical use. That AnSCs (Rong et al. 2020), AnSC-conditioned medium (Rong et al. 2019) and AnSC-Exosomes (Zhang et al. 2023a) achieved similar results on stimulating regenerative wound healing indirectly supports the claim that the effects of PP on regenerative wound healing over a pedicle stump must have been through the long range of the paracrine pathway.

No matter which pathway the mesenchymal stem cell conditioned may take to effect wound healing, an approach using conditioned medium for treating wounds in the clinic could help reduce the biological variability of cell-based therapy, overcoming concerns about cell origin and immuno-compatibility and allowing more precise dosing with purified paracrine components. This approach could facilitate the development of safe and effective cell-free regenerative reagents with more predictable and controllable outcomes. The disadvantages for the deer antler case are that the paracrine factors from AnSCs are extremely limited in quantity and alter the healing skin to velvet type.

Interestingly, at the initial stage of antler regeneration in an adult deer, the process of wound healing over the top of a pedicle stump closely resembles that of fetal skin (Guo et al. 2021). In the wound healing over a pedicle stump, there is essentially no contraction being detectable, as the pedicle skin and the enveloped bone are intimately associated with each other without interposition by a layer of subcutaneous loose connective tissue (Li and Suttie 2000). The rate of healing observed in the deer antler is extremely rapid: a wound of 10 cm in diameter can heal within 10 days, and the nature of the healing is regenerative (Li et al. 2005). At the morphological and histological levels, the initial stage of antler regeneration being achieved by way of a regenerative wound healing mechanism including de novo development of skin appendages is well-described (Guo et al. 2021). Consequently, we believe that AnSCs have likely converted the phenotypes of adult fibroblasts in the healing dermis into fetal-like phenotypes through paracrine influences which have facilitated the regenerative wound healing.

## Systemic factors alone stimulate regenerative wound healing without altering skin type, are seasonal-specific but not species-specific

The phenomenon that removal of PP results in failure of antler regeneration, but does not inhibit initial regenerative wound healing by pedicle skin-type (Li et al. 2007), indicates that systemic factors, possibly from circulating blood, alone have the capability of stimulating generic regenerative wound healing. Over the years of working with deer, we noticed that cutaneous wounds that either accidentally occurred or resulted from surgery on deer can heal not only quickly but also in a regenerative manner if in summer during the antler growth period; but slow and in a scar healing manner in winter. This observed phenomenon is further supported by our recent experimental results, in that healing speed of the artificially induced (by castration) pedicle wound in winter (Bubenik 1982) was less than half of that in summer, and healing quality was also inferior (Guo et al. 2025).

**Fig. 4:**
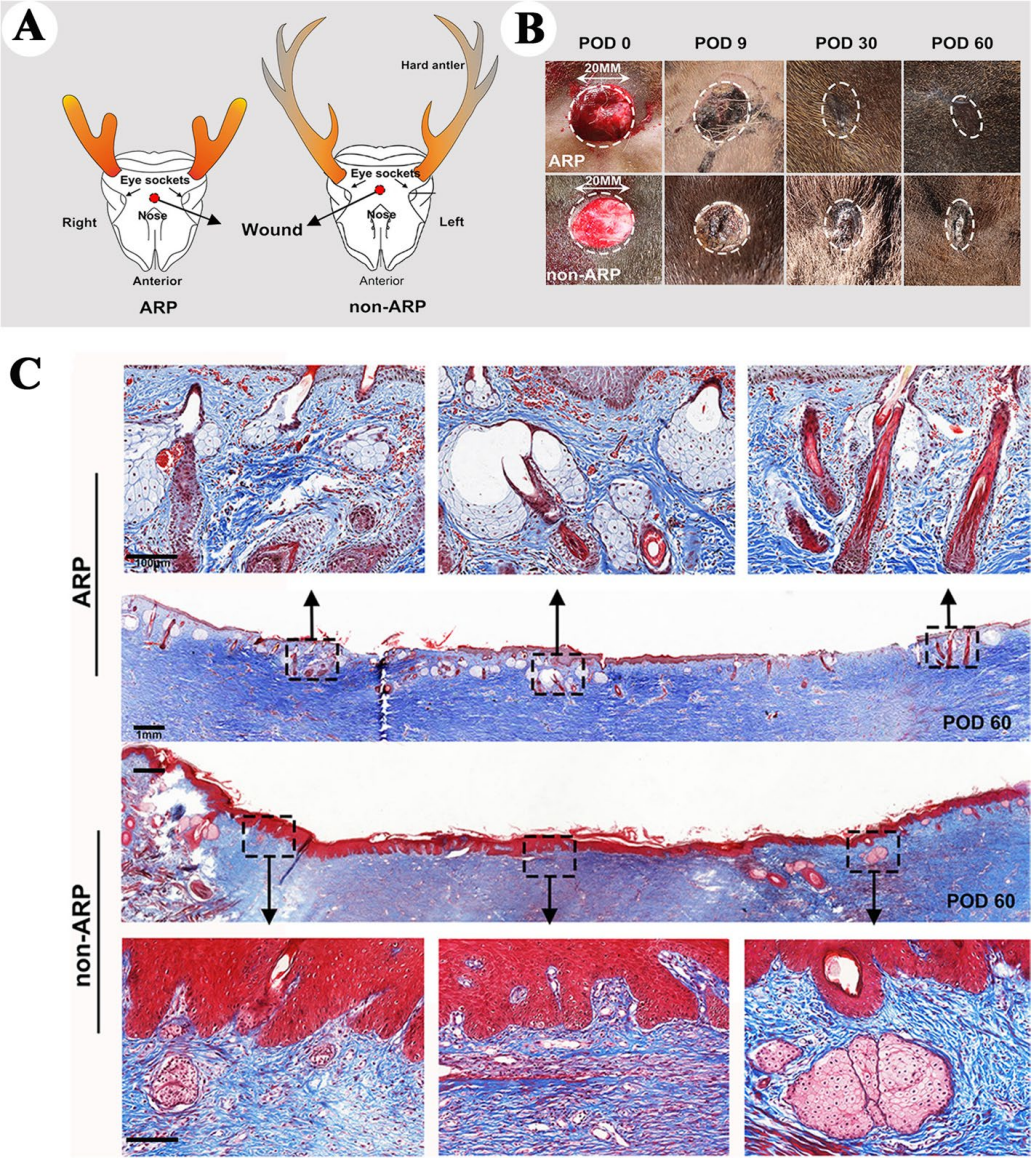
Macroscopic and microscopic examinations of forehead skin wound healing both in ARP and in non-ARP in adult sika deer (Reproduced with the permission from Fig. 1; npj Regenerative Medicine; 10.1038/s41536-025-00391-5). **A**. Schematic drawing to show wounding sites in ARP and in non-ARP. **B**. Macroscopic examination of the healing process of FTE wounds on the foreheads of sika deer on post operation days (POD) 0, 9, 30, or 60. The healing skin grew centripetally, encircling the scab. Note that on POD 30, healing was morphologically completed both in the ARP-wounds and in non-ARP-wounds. However, the former was healed in a regenerative manner and the latter in a scar manner. **C**. Tissue sections of healed skin on POD 60 in FTE wounds both in ARP and non-ARP. Note that numerous neogenic appendages were visible in the ARP-wounds, but not in the non-ARP-wounds

To confirm this postulation, very recently we devised a way through which systemic factors are effectively teased apart from local factors, i.e., creation of FTE wounds on the deer forehead region, a place distant away from the pedicle (Fig. 4A), in ARP (June) and non-ARP (September). Notably, all these wounds created in ARP were healed in a regenerative manner largely by the regenerated forehead skin (Fig. 4B upper), but in non-ARP healed in a scar manner (Fig. 4B, lower). Histological results showed that wound healing in ARP belongs to the regenerative type, which contains different developmental stage hair follicles, sebaceous glands and sweat glands; whereas the outcome of the healing in non-ARP is scar tissue, which contains no skin appendages but thick collagen bundles (Fig. 4C). In addition, compared to the wounds on the deer foreheads in non-ARP, the overall course of wound healing in ARP had significant less leukocytes influx, indicating less immune reaction is involved in the ARP wound healing. Therefore, systemic factors in ARP in summer alone are capable of inducing regenerative wound healing and this healing does not involve an alteration of skin type to antler velvet (Guo et al. 2025).

**Fig. 5:**
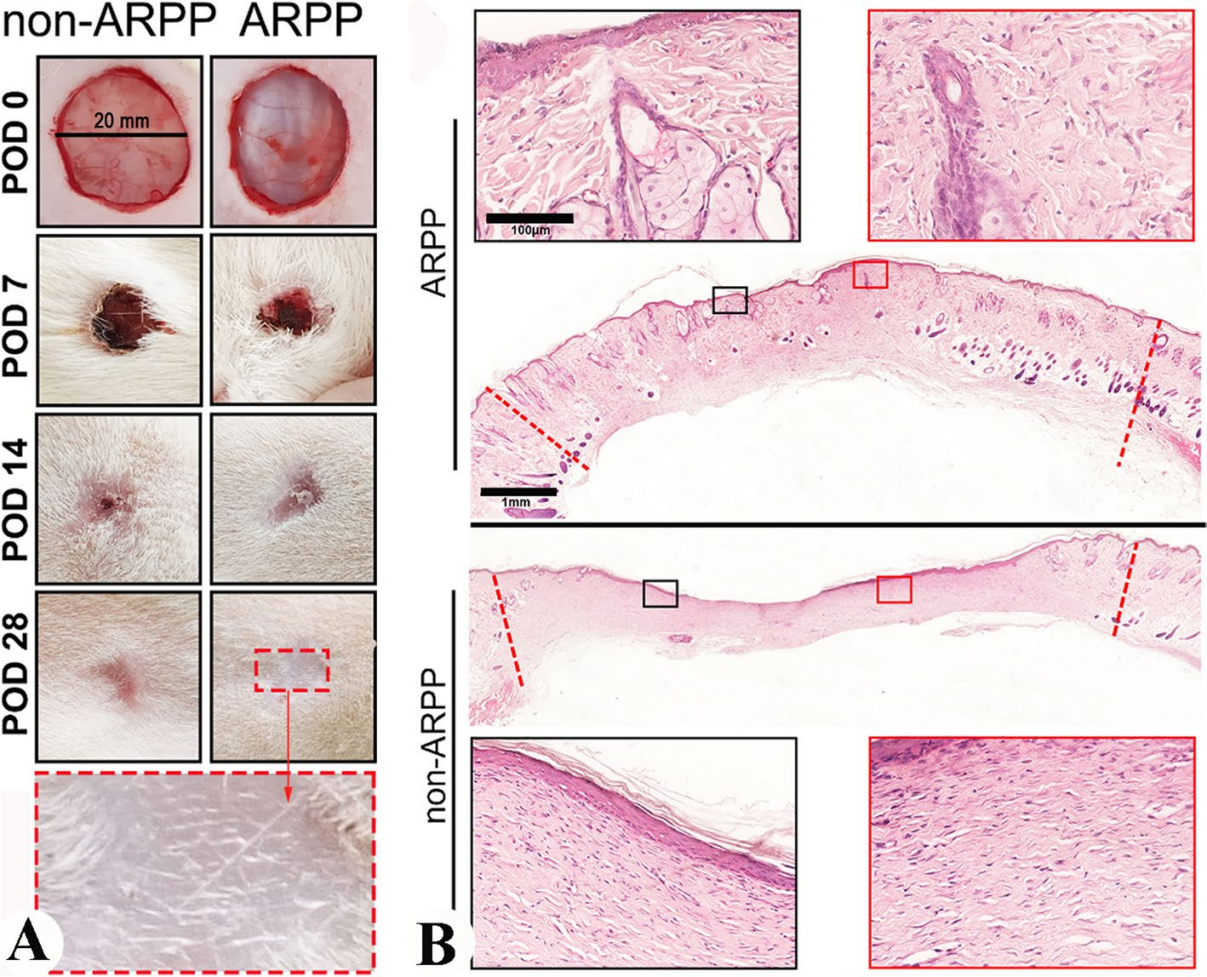
Morphological and histological examination of FTE wound healing in rats being treated with systemic factors from ARP or from non-ARP (Reproduced from the permission from Fig. 4b and c; npj Regenerative Medicine; 10.1038/s41536-025-00391-5).** A**. Macroscopic examinations of the wound healing course. Note that on POD 28 the wounds in both treatment groups were essentially completed, but only those treated with systemic factors from ARP showed scar-less (inset). **B**. Histological sections. The healed wound tissue treated with systemic factors from ARP contained numerous skin appendages (upper, insets); whereas no skin appendage was detected from non-ARP (lower, insets)

Whether or not the effects of systemic factors on regenerative wound healing are worth further pursuing solely depends on whether these effects are species-specific or can also apply to other mammalian species including humans. Given that local factors from AnSCs are not species-specific (Guo et al. 2021; Rong et al. 2020; Zhang et al. 2023a), we assumed that systemic factors from deer blood in ARP may be likewise. Therefore, we investigated this hypothesis using FTE wounds again in a rat model. Topical application of freeze-dried deer blood plasma in hydrogel (ARPP; source of systemic factors collected in ARP) on rat wounds promoted regenerative healing significantly compared to the controls of no-treatment or treatment with plasma from non-ARP (non-ARPP; Fig. 5), suggesting these systemic factors are not species-specific (Guo et al. 2025). Again, ARPP-treated wounds in rats exhibited a significant less severe infiltration of leukocytes into the wound bed during the process of healing, whereas non-ARPBP-treated wounds showed much stronger immune reaction; thus, much less immune response was involved in ARPP treated wounds. This finding offers the potential to develop a cell-free therapeutic for cutaneous wound healing in the clinic setting.

## Novel systemic factors are identifiable and isolatable

To identify the putative novel factors for regenerative wound healing in deer blood plasma (ARPP) collected in summer, we conducted comparative analysis through DIA quantitative proteomics between the ARPP and non-ARPP. The results showed that the differentially up-regulated proteins in ARPP were mostly related to wound healing or tissue regeneration, such as IGF1, PRG4, MMP2, POSTN, ADIPOQ, etc., and these factors must be the ones that created a pro-regenerative environment in ARP. The finding of a high level of IGF1 in ARPP is consistent with the report that circulating IGF1 levels are significantly elevated during antler growing season relative to the other seasons and show a strong positive correlation with antler growth rate (Suttie et al. 1985, 1988b). A high level of IGF1 likely contributes to the enhanced effects on cell proliferation in the healing tissues either in deer or in rat in our studies. Surprisingly, proteoglycan 4 (PRG4) was found to be also included in the highly up-regulated factors in a study by Guo et al. (2025). PRG4 is synthesized by chondrocytes located at the surface of articular cartilage and by some synovial lining cells. This protein contains both chondroitin sulfate and keratin sulfate glycosaminoglycans (Schmidt et al. 2004), and has the functions of reducing friction between articulating surfaces (e.g. cartilage). PRG4 is also reported to be highly expressed in regenerating antler cartilage tissue (Sunwoo et al. 1998). Here, we unexpectedly found that PRG4 has the potential to enhance cutaneous wound closure and promote regenerative wound healing.

**Fig. 6:**
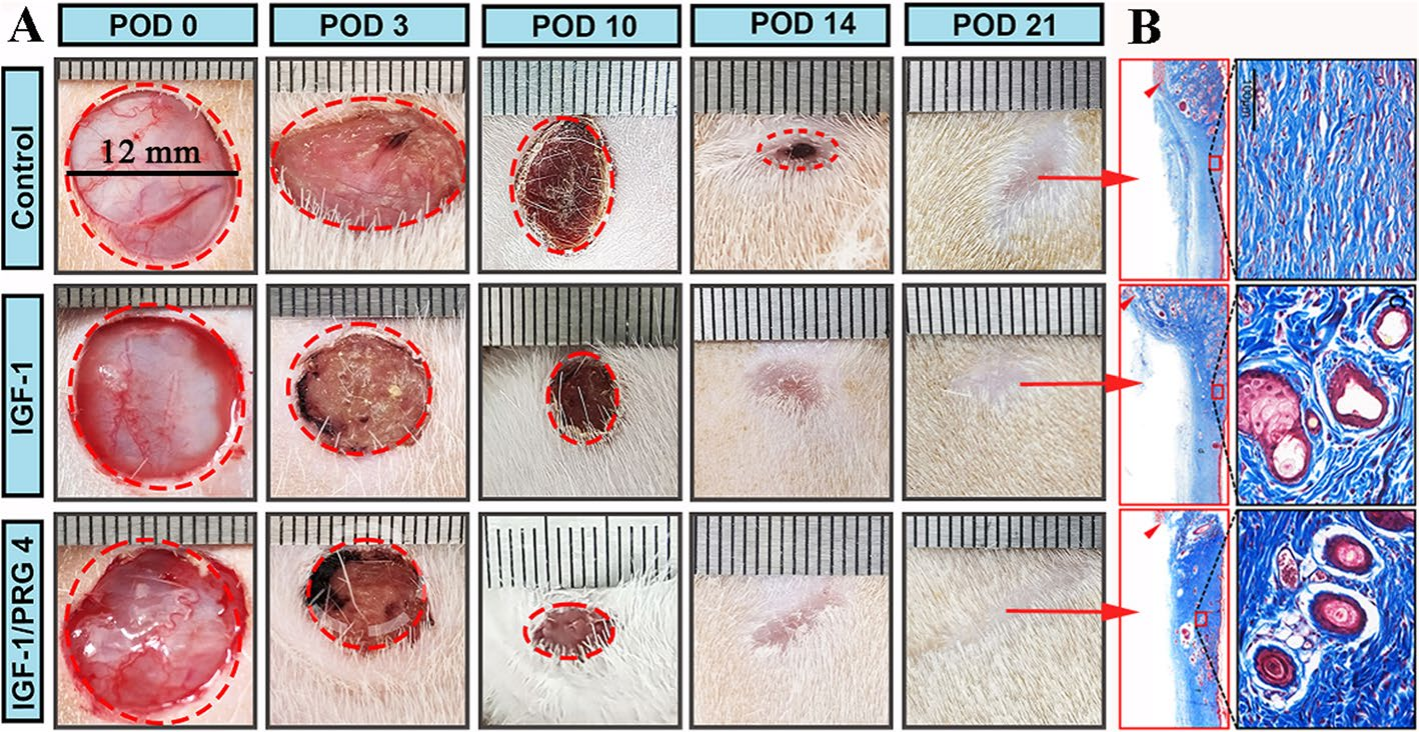
Effects of substances, IGF1 or IGF1 + PRG4, from the blood plasma collected in ARP (ARPP) on rat FTE wound healing (Reproduced with permission from Fig. 9a and 9c; npj Regenerative Medicine; https://doi.org/10.1038/s41536-025-00391-5). **A**. Macroscopic examinations of the wound healing course. Note that on POD 14, wound healing in the IGF1 + PRG4-treated group was essentially completed, in the IGF1-treated group had reached final stage, but in the control group was in the mid healing stage (circle of red broken-line). **B**. Histological sections. Note that the healed skin tissue treated with either IGF1 or IGF1 + PRG4 contained numerous skin appendages, and the latter had even more appendages than the former. Whereas no skin appendage was detected in the control group

When IGF1 was combined with PRG4 on FTE wounds using the same rat model above, we found that the effects of the combination not only improved wound healing significantly, including accelerated healing speed and increased formation of skin appendages, but it also had a better therapeutic outcome than IGF- 1 alone (Fig. 6). Besides these two, other factors with high expression levels in ARPP have also been reported to be related to the promotion of tissue regeneration (Table S1) and may have combined action on regenerative restoration, thus worth further exploitation.

## Exploration of the mechanism underlying the systemic factor stimulation of regenerative wound healing

Wound healing is generally divided into three distinct but overlapping phases: inflammation, proliferation, and remodeling (Baron et al. 2020). To reveal the underlying mechanism of the regenerative outcome induced by systemic factors in ARPP, we investigated the major healing hallmarks in these three phases (Guo et al. 2025). In brief, in the inflammation phase, ARPP treatment enhanced an anti-inflammatory but suppressed a pro-inflammatory response; in the proliferation phase, it inhibited myofibroblast differentiation, but increased the ratio of TGF-β3/TGF-β1; and in the remodeling phase, it induced ECM regeneration through orchestrating collagen remodeling. These unique characteristics are consistent with those observed in the regenerative healing of deer forehead skin in the same study, suggesting that systemic factors in ARPP likely induce regenerative wound healing through these unique molecular events and is not species-specific.

The biggest contrast in blood plasma composition between ARPP and non-ARPP would be the concentrations of androgen hormones and IGF1, based on the previous studies. It is known that testosterone levels in circulating blood in ARP in summer is at its nadir (< 1 ng/ml); whereas it is at its highest (> 10 ng/ml) in non-ARP in autumn rutting season (Bubenik 1982; Suttie et al. 1995). As opposed to androgen hormone levels, IGF1 concentration in ARP is at its highest plateau in ARP and lowest in non-ARP (Suttie et al. 1985, 1988a). Androgen has a notorious reputation for retardation of cutaneous wound healing. Ashcroft and Mills (2002) found that castration of male mice results in a striking acceleration of local cutaneous wound healing, which is associated with a reduced inflammatory response via direct downregulation of pro-inflammatory cytokine expression by macrophages and increased hair growth. Decrease in testosterone level by castration or blockade of androgen action via receptor antagonism stimulates the healing response not only through hair follicle epithelial/mesenchymal cell proliferation, but directly via effects on wound cell populations. Gilliver et al. (2007) reported that wound deposition of both type I collagen and fibronectin is increased in castrated male rats compared with the controls. This response is accompanied by overall decreases in the levels of key collagenolytic enzymes, mainly matrix metalloproteinases, rather than by increase in synthesis of extracellular matrix. Therefore, increased wound collagen deposition in androgen-deprived rats must result from reduced matrix degradation, rather than enhanced matrix protein biosynthesis.

IGF1 is a generic mitogen capable of stimulating DNA synthesis, cell proliferation, protein synthesis, glucose transport, and secretion of glycosaminoglycans and proteoglycans by dermal fibroblasts (Achar et al. 2014). In wound healing, IGF1 was detected in the wound tissue fluid of rats and pigs (Marikovsky et al. 1996; Steenfos and Jansson 1992). Achar et al. (2014) reported that all epidermal cells, macrophages and other cell types express IGF1 at 1 to 3 days after the injury; IGF1 directly participates in the cellular granulation process and stimulates more rapid re-epithelialization of wounds. Our result (Guo et al. 2025) that IGF1 levels in ARPP were significantly higher than in non-ARPP is consistent with the currently held view and the report by Suttie et al. (1985).

It is reported that endocrine mechanisms underlying wound healing are mainly associated with modulations of TGF-β and α-SMA production (Meng et al. 2016). Expression of these molecular effectors can stimulate ECM production, fibroblast proliferation, cell migration and angiogenesis (Marangoni et al. 2015; Monika et al. 2021). The TGF-β family has been shown to be closely involved in either regenerative healing or scar formation. TGF-β3 (so-called anti-fibrotic isoform) is more abundant than TGF-β1 (so-called fibrotic isoform) in fetal wounds (Kishi et al. 2012). In fetal fibroblasts, the levels of TGF-β1 are downregulated, whereas the levels of TGFβ3 are upregulated (Walraven et al. 2014; Satish and Kathju 2010). TGF-β1 stimulates more fibroblasts to transform to myofibroblasts (α-SMA as a principal marker), which are major players in wound contraction and the cell type that secretes type I collagen (Chen et al. 2014). In one of our previous studies, we treated FTE wounds in the rat model with growing antler extracts (contains mainly systemic factors) and found that expression of TGF-β1 in the healed tissue was downregulated significantly, but TGF-β3 was upregulated significantly (Zhang et al. 2023b), which makes the ratio of TGF-β3/TGF-β1 closely resembles that of fetal skin.

In the same study, we also detected levels of different types of collagens in the healed skin (Zhang et al. 2023b). It is reported that fetal fibroblasts differ from adult fibroblasts in collagen synthesis in terms of speed of deposition, variation in collagen type ratios and quantity of collagen. Most striking is the persistence of excess Col 3 over Col 1, with healed wounds in the fetus showing ratios around 3:1 of Col 3/Col 1, instead of the 1:3 ratio observed in adult healed wounds (Merkel et al. 1988). Chen et al. (2014) have shown that higher levels of Col 3 yield smaller, reticular structures with more cross-linking than Col 1, and contributes towards scarless wound healing. In our study (Zhang et al. 2023b), we found that the ratio of Col 3 A1/Col 1 A2 in healed tissue in the AnSC-CM treated group could match that of fetal skin. Therefore, it is likely that systemic factors in ARPP capable of stimulating regenerative wound healing may do so via creation of a fetal-like environment that favors scar-less wound healing.

## Unique wound healing phenomenon: wound is held in abeyance for more than half a year

Besides rapid regenerative healing, wounds around/over a pedicle have another unique feature, namely, once created, these wounds are held in abeyance without healing for over half a year (Goss 1983). In the late summer or early autumn, sharply increased testosterone fully calcifies growing antlers and totally occludes blood supply, which kills velvet skin and causes it to shed, then a broken wound edge of pedicle skin around the base of the hard antler is created (Fig. S2A, B). However, these wounds remain quiescent until the next spring when the level of circulating testosterone decreases to a certain threshold (> 1.0 ng/ml) which triggers the hard antler to cast and wound healing then follows (Suttie et al. 1995; Li et al. 2009a, b). During this seven-months-odd dormant period, these wounds do not exhibit infection, inflammation or ulceration (Guo et al. 2021). Here one may argue that the persistent hard antler may be in the way to prevent this integumental healing in the first place. However, this is not the case, because even if the hard antler or even including distal part of the pedicle is removed at this stage, its bone still remains exposed in the absence of centripetal healing by the surrounding skin (Goss 1995). Conversely, if the hard antler/button fails to cast for some reason, wound healing still takes place in the antler regeneration season (spring). If that is to happen, the hard antler/button would be wrapped by the healing skin and a so called “double head” (Kierdorf and Kierdorf 1992) will be formed (Fig. S2C, D). In some deer species, such as white-tailed deer (Fig. S2E), the hard antlers drop off in midwinter, while the pedicle skin wound remains quiescent (Fig. S2F) until the coming spring, a period over 3 months (Goss 1983; Ullrey 1982).

Wound healing is one of nature's processes, which is extremely hard to stop experimentally. Yet in the case of deer antlers, when the velvet is shed, the broken wound edges of pedicle skin are created and these wounds remain dormant and discontinuous. Not until the spring comes does the healing reaction occur. Up to now, we do not know whether dormancy of the pedicle skin wounds is caused by local factors, systemic factors or both, although it is clear that hard antler casting is triggered by a decrease in circulating testosterone. We believe that the phenomenon of wound healing in abeyance is likely caused by local factors as cutaneous wounds on the deer body in winter also heal, albeit significantly slower than in any other seasons (26). A simple experiment should tell one way or the other. In the experiment, creation of a wound, with similar size to the fresh pedicle wound formed following hard antler casting, on the forehead region (distant away from the local factors) of a male white-tailed deer. Subsequently, to observe how the wound healing is to proceed. If the forehead wound is also held in abeyance, systemic factors must be responsible for the dormancy of the wound healing, otherwise local factors would have to be the main player for this phenomenon.

Irrespective of which way, revealing the underlying mechanism can not only make a contribution to the fundamental understanding of wound healing per se, but also have implications for helping to treat certain clinical situations, for example, development of specialized orthopedic prostheses for amputees. If we can reveal the mechanism underlying the phenomenon that pedicle wounds are held in abeyance peacefully for a seven-month period, we would be able to develop a device in which the cutaneous wounds resulting from a percutaneous prosthesis would remain firmly and peacefully attached to the artificial gear relatively permanently without suffering chronic inflammation and ulceration. This idea has been successfully verified by Pendegrass et al. (2006), in which they fabricated orthopedic prostheses by mimicking roughness of pedicle bone surface and effectively solved the problem of soft tissue attachment.

## Conclusions

**Fig. 7:**
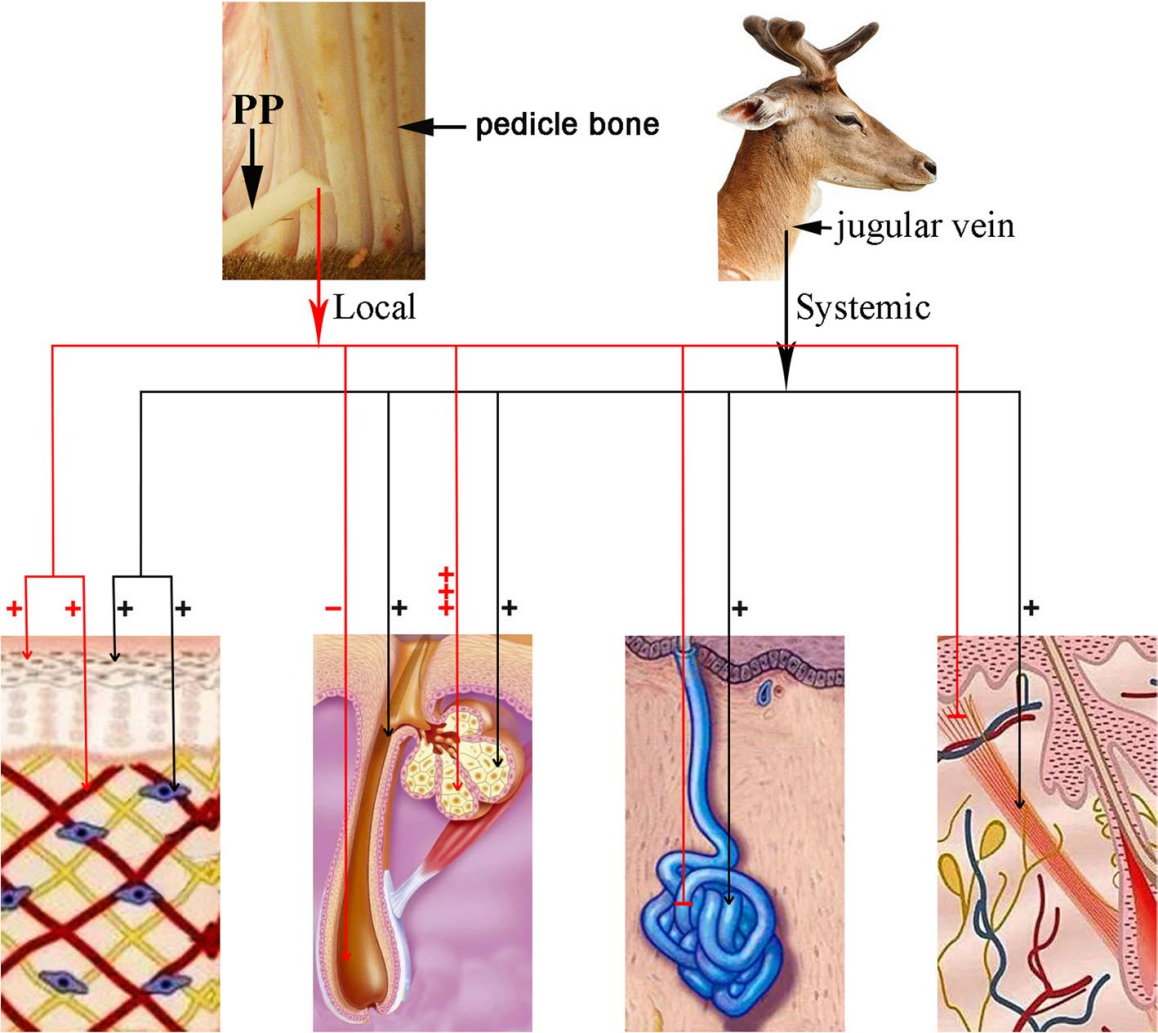
Schematic illustration of effects of local and systemic factors on regenerative wound healing over a pedicle stump during initiation stage of antler regeneration. This regenerative healing is regulated by both local and systemic factors. The former plays roles in promoting healing cell proliferation, altering healing skin type (from scalp to velvet), miniaturizing hair follicles, producing large and multi-lobed sebaceous glands and terminating sweat glands and arrect pilli muscle; the latter promotes a generic regenerative cutaneous wound healing including cell proliferation in the healing tissue and formation of general appendages of somatic skin

Overall, regenerative wound healing over a pedicle stump prior to antler regeneration is regulated by both local and systemic factors. The former plays indispensable roles in antler regeneration per se and in alteration of healing skin type from pedicle type to velvet skin, including miniaturizing hair follicles, producing large and multi-lobed sebaceous glands and terminating sweat glands; the latter promotes generic wound healing, including enhancing an anti-inflammatory and suppressing a pro-inflammatory response, inhibiting myofibroblast transition, increasing the ratio of TGF-β3/TGF-β1, and orchestrating collagen remodeling (Fig. 7). These findings undoubtedly have medical applications: 1) through analyzing the local factors, we may identify the molecules/substances that are specifically responsible for regulating the formation of each type of skin appendage, such as what factors impede hair follicles, exaggerate sebaceous glands and inhibit sweat glands? 2) Discovering the role of systemic factors in generic regenerative wound healing would have essentially identified an almost unlimited source for clinic use, and further enrichment of the deer blood plasma for key effective factors would greatly enhance their efficacy on regenerative wound healing in general.

## Supplementary Information

Supplementary Material 1. Fig. S1. Characterization of the healed skin of FTE wounds in rats with different treatments. HE staining. Fig. S2 Static state of pedicle wound healing for an exceedingly long period. Table S1. Information of the relevant factors in wound healing identified from ARPP.

## Data Availability

Not applicable.

## Abbreviations

ADIPOQ: Adiponectin, C1Q, and collagen domain
AnSCs: Antler stem cells
AP: Antlerogenic periosteum
ARP: Antler regeneration period
ARPP: Plasma from antler regeneration period
CM: Conditioned medium
Col: Collagen
ECM: Extracellular matrix
α-SMA: α-Smooth muscle actin
FTE: Full-thickness excision
hMSC: Human-mesenchymal stem cell
IGF1: Insulin growth factor 1
MSCs: Mesenchymal stem cell
MMP2: Metalloproteinase 2
RM: Reserve mesenchyme
POSTN: Periostin
PP: Pedicle periosteum
PRG4: Proteoglycan 4
TGF-β: Transforming growth factor-β
