# Supplementary material for: Local and systemic factors both required for full renewal of deer antlers, and systemic factors only for generic cutaneous regenerative healing
Source: Cell Regen. 2025 Jun 10;14:24. doi: 10.1186/s13619-025-00233-1 (PMC12151924; doi:10.1186/s13619-025-00233-1)
Supplement: Supplementary file 1 — Supplementary Material 1. Fig. S1. Characterization of the healed skin of FTE wounds in rats with different treatments. HE staining. Fig. S2 Static state of pedicle wound healing for an exceedingly long period. Table S1. Information of the relevant factors in wound healing identified from ARPP. [file 13619_2025_233_MOESM1_ESM.docx]

Supplemental Materials


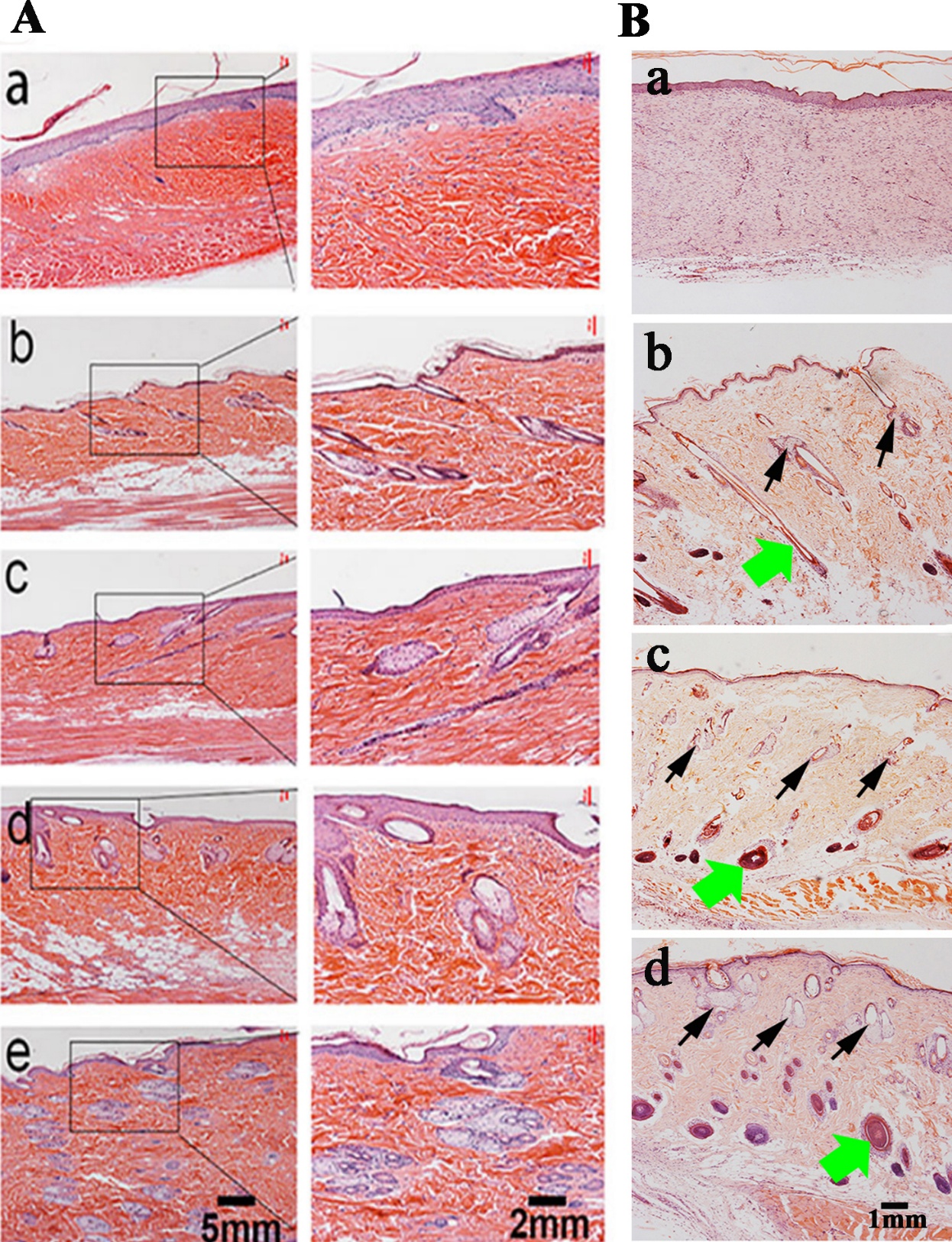


Fig. S1 Characterization of the healed skin of FTE wounds in rats with different treatments. HE staining.

1. Histological sections of the healed skin (Reproduced with the permission from Fig. 4A; Cell Transplantation; <https://doi.org/10.1177/0963689720951549>). **a**, no treatment in model group; **b**, intact; **c**, human stem cells; **d**. rat stem cells; **e**, AnSCs. Note that regenerative wound healing was occurred in all three groups with stem cell treatment, but sebaceous glands were exaggerated and densely populated only in the AnSC groups (**e**).
2. Histological sections of healed skin (Reproduced with the permission from Fig. 6a; Stem Cell Research & Therapy; <https://doi.org/10.1186/s13287-019-1457-9>). **a**, no treatment in the model group; **b**, EGF; **c**, MSC-CM; **d**, AnSC-CM. Note that only AnSC-CM contained more and exaggerated sebaceous glands (black arrow). Green arrow: hair follicles.


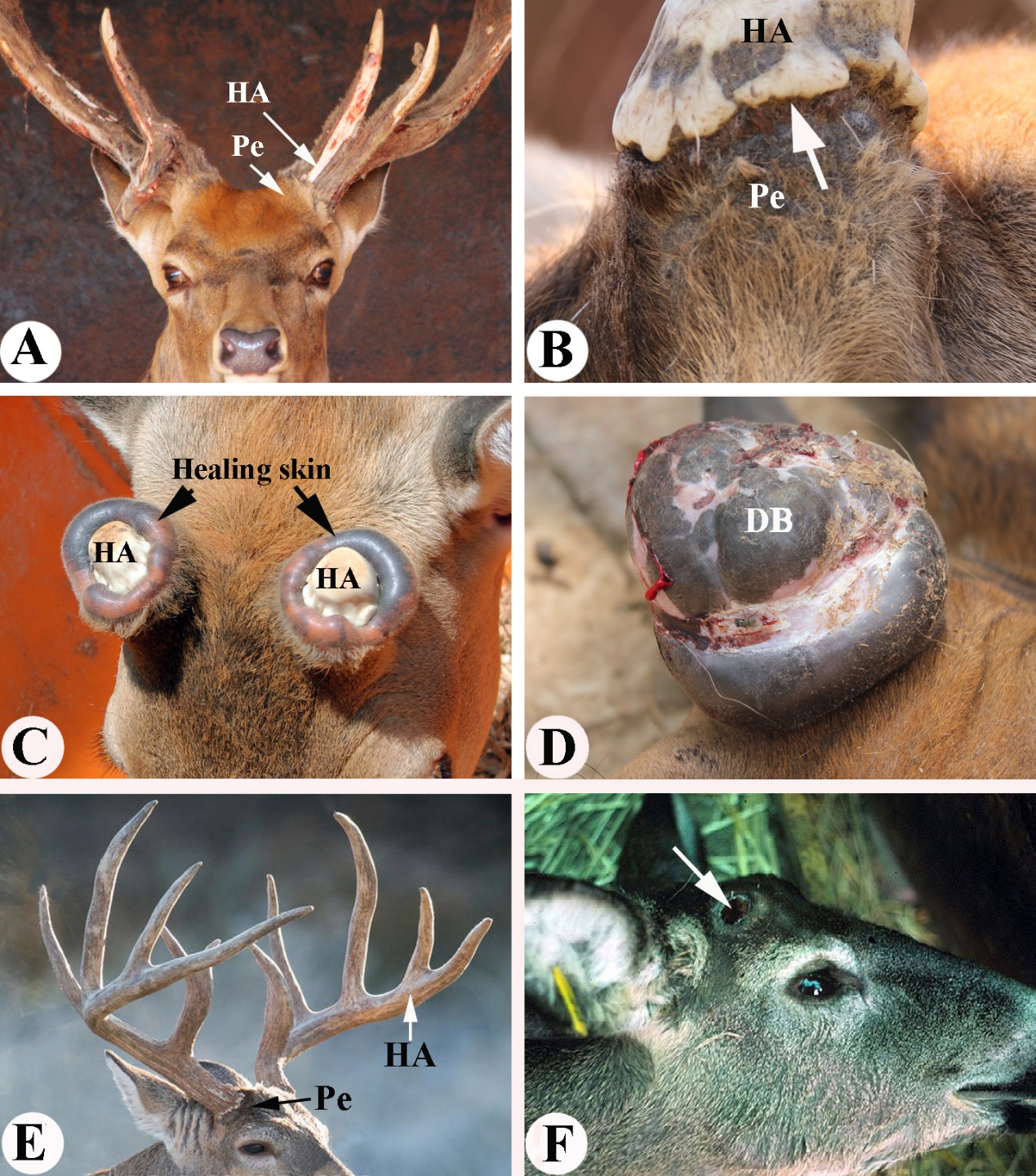


Fig. S2 Static state of pedicle wound healing for an exceedingly long period.

1. Velvet skin shedding and creation of the broken wound edges of pedicle skin in sika deer in autumn. Note wound margin is located at the junction between hard antler (HA) and pedicle (Pe).
2. Hard antler (HA) and pedicle (Pe) in sika deer in winter. Note the distal end of broken edge of pedicle cutaneous wound is just located beneath the hard antler base (arrow).
3. Double-head formation in sika deer. Naturally, pedicle healing sequentially follows the hard antler casting. However, if for some reason hard antlers/buttons fail to cast on time, pedicle wound healing will still take place. If that is to happen, the hard antler/button (HA) will be wrapped by the healing velvet skin (arrows), then a so-called “double-head” will form.
4. A typical double-head, in which a regenerating antler fully wraps the un-cast hard button (HB) in sika deer.
5. A white-tailed deer stag in early winter. Note that the stag was carrying a pair of hard multi-branched antlers (HA) on its short pedicles (Pe).
6. A static state pedicle wound (arrow) created following the hard antler casting in mid-winter in white-tailed deer. This wound is held in abeyance for at least three months before commencement of wound healing process.

Table S1. Information of the relevant factors in wound healing identified from ARPP


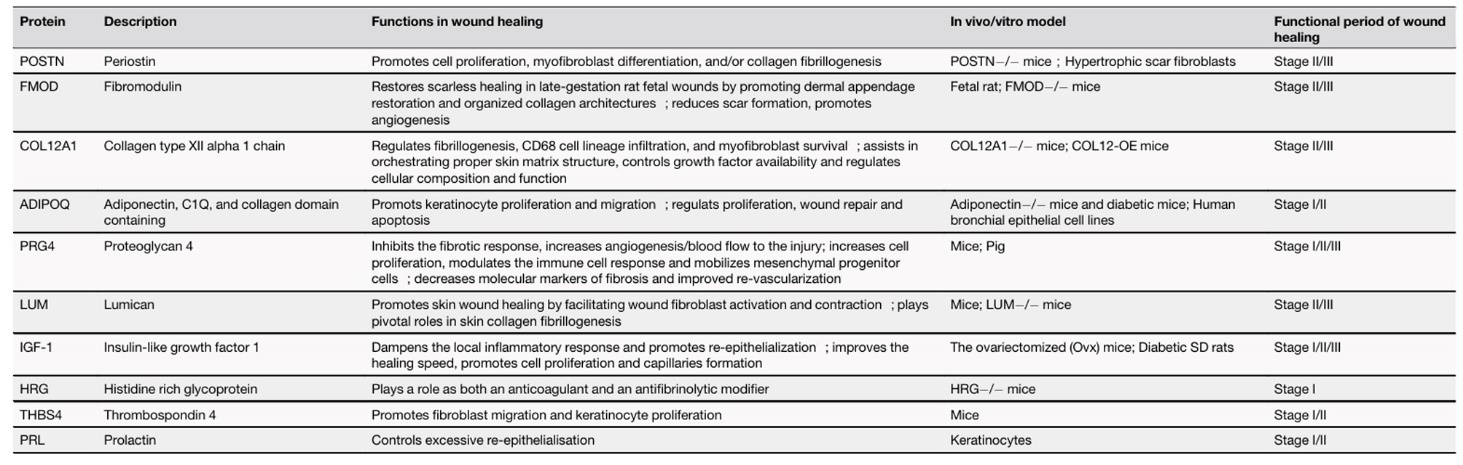


^🞹^Reproduced with the permission from Table 1; npj Regenerative Medicine; https://doi.org/10.1038/s41536-025-00391-5.
